# 513. Use of Antimicrobial Stewardship Program in Operationalizing Monoclonal Antibody Therapy in SARS-CoV-2 Infection

**DOI:** 10.1093/ofid/ofab466.712

**Published:** 2021-12-04

**Authors:** Jessica Abrantes-Figueiredo, Stephanie Nalewyko, Dora E Wiskirchen

**Affiliations:** Saint Francis Hospital and Medical Center, Hartford, Connecticut

## Abstract

**Background:**

Antimicrobial stewardship programs (ASP) have been essential during the coronavirus disease 2019 (COVID-19) pandemic response. Use of monoclonal antibodies for severe acute respiratory syndrome coronavirus 2 (SARS-CoV-2) infection has proven difficult to operationalize, despite being available through emergency use authorization (EUA). Utilizing existing ASP and multidisciplinary approach to lead the effort, we aim to describe our experience in operationalizing monoclonal antibody therapy.

**Methods:**

Retrospective study of SARS-CoV-2 infected adults receiving monoclonal antibody therapy under EUA (December 2020-April 2021). An algorithm developed by the ASP provided education and an interactive online tool allowing referring physicians and patients to assess eligibility prior to hospital arrival. Patients were screened and approved by existing ASP which included; Infectious Disease (ID) physicians, pharmacist, and ID Nurse. A multidisciplinary approach with ER staff and development of pharmacy workflow with order set were utilized as eligible patients received infusion in dedicated ER location. Data such as demographics, co-morbid condition, infusion related complications, hospitalization, and death were reviewed and collected regularly by the ASP team with frequent monitoring and regulatory reporting. Primary patient outcome was preventing hospitalization.

**Results:**

107 patients received monoclonal antibody therapy. 47% patients were male, 50% White, and 79% non-Hispanic. 87% received monotherapy (bamlanivimab) and 13% received dual therapy (bamlanivimab/etesevimab). 17 patients required hospitalization post infusion. 1 death occurred. COVID-19 related hospitalization within 30-days was avoided in 84% of treated patients. No adverse event directly related to infusion were seen.

**Conclusion:**

Use of monoclonal antibody therapy under EUA for patients for SARS-CoV-2 infection led to decrease in hospitalization in this cohort. An existing ASP using an algorithmic approval process, frequent monitoring, and multidisciplinary approach successfully operationalized the use of monoclonal antibody therapy. ASP’s provide benefit and versatility beyond monitoring of antimicrobials alone and should continue to receive support by hospital leadership.

**Disclosures:**

**All Authors**: No reported disclosures

